# Prophylactic intra-aortic balloon pump in patients with left main disease undergoing off-pump coronary artery bypass grafting

**DOI:** 10.1186/s12872-020-01554-6

**Published:** 2020-06-03

**Authors:** Ju-Bing Zheng, Kun Hua, Kui Zhang, Shao-You Zhou, Shi-Jun Xu, Juan-Juan Sheng, Ran Dong

**Affiliations:** Department of Cardiac Surgery, Beijing Anzhen Hospital, Capital Medical University, No. 2 Anzhen Street, Chaoyang District, Beijing, 100029 China

**Keywords:** Left Main disease, Off-pump coronary artery bypass grafting, Intra-aortic balloon pump

## Abstract

**Background:**

Preventive intra-aortic balloon pump (IABP) for high-risk patients with stable hemodynamics is controversial, and its definition of high-risk is still unclear. This study aimed to investigate the effect of prophylactic IABP on the early outcome of left main disease (LMD) patients receiving off-pump coronary artery bypass grafting (OPCABG) with stable hemodynamics.

**Methods:**

From January 2013 to April 2020, 257 consecutive patients who underwent OPCABG through sternotomy were enrolled in this study. All LMD patients (greater than 70%) had stable hemodynamics (BP>100 mmHg without vasoconstrictor substance infusion). Early outcomes of 125 patients with prophylactic IABP (IABP group) and 132 patients without IABP (Control group) were compared in this study.

**Results:**

IABP did not show favorable effect on the conversion to CPB (RR 0.63, 95%CI 0.05–7.89, *P* = 0.7211), perioperative MI (RR 0.69, 95%CI 0.22–2.12, *P* = 0.5163), mortality (RR 0.65, 95%CI 0.04–10.25, *P* = 0.7608) or the composite end of the conversion, MI and mortality (RR 0.63, 95%CI 0.23–1.74, *P* = 0.3747). There was greater incidence of prolonged ventilation in IABP after adjustment (RR2.16, 95%CI 1.12–4.18, *P* = 0.0221). There was no IABP-related mortality or limb ischemia.

**Conclusion:**

No significant difference in early outcomes was observed in hemodynamically stable patients with LMD between prophylactic IABP group and control group. Prophylactic IABP may be unnecessary in patients with LMD undergoing OPCABG.

## Background

Intra-aortic Balloon Pump (IABP) is the most commonly used circulatory assist device in the treatment of intractable angina, cardiogenic shock or heart failure, and complications of myocardial infarction. It could provide more favorable myocardial blood supply by increasing diastolic pressure [1, 2]. In recent years, preoperative prophylactic IABP for high-risk patients with stable hemodynamics has been reported and there is controversy about this [3–7]. In addition, the definition of high risks is unclear because it is usually a combination of at least 2 of the following risk factors: poor ejection fraction (EF), advanced age, presence of mitral regurgitation, left main coronary artery disease, redo coronary artery bypass grafting (CABG), New York Heart Association (NYHA) class III or IV, urgent or emergent CABG, and left ventricular end diastolic pressure > 20 mmHg etc. [8, 9]. However, report of prophylactic IABP in patients with left main artery disease (LMD) who underwent off-pump coronary artery bypass grafting (OPCABG) remains limited. In addition, neither off-pump coronary artery bypass grafting (OPCAB) or on-pump CABG in these studies has evaluated the stratification of IABP. The role of preventive IABP in isolated LMD patients receiving OPCAB is unclear. The purpose of this study was to investigate the effect of prophylactic IABP on the early outcome of LMD patients receiving OPCABG with stable hemodynamics.

## Methods

### Patients

From January 2013 to April 2020, 257 consecutive patients who underwent OPCABG through sternotomy were enrolled in this study. All LMD patients (greater than 70%) had stable hemodynamics (BP>100 mmHg without vasoconstrictor substance infusion).

Exclusion criteria: patients undergoing emergency or urgent operations due to unstable hemodynamics or intractable angina; patients with mechanical complications due to acute myocardial infarction; patients with poorly preoperative ventricular arrhythmias control; patients with myocardial infarction within 3 days; patients with unstable angina due to persistent myocardial infarction. Early outcomes of 125 patients with prophylactic IABP (IABP group) and 132 patients without IABP (Control group) were compared in this study. The longest follow-up time was 1 year. This study was approved by the Beijing Anzhen Hospital Ethic Committee. Informed consent was obtained from all individual participants included in the study.

### IABP insertion and timing

One hundred twenty five patients received prophylactic IABP support according to the decision of surgeons. Intra-aortic balloon was percutaneous inserted into the common femoral artery 30 min before the operation. After IABP insertion, all patients were anticoagulated with heparin to maintain the activated partial thromboplastin time 1.5–2 times the normal value. In all patients receiving IABP insertion, 9.5F Percor balloon was percutaneous inserted into the common femoral artery using 10F sheath.

### Surgical procedure

All procedures were performed through a median sternotomy. After left internal mammary artery (LIMA) and saphenous vein (SV) were harvested, heparin was injected into the patient to keep an activated clotting time above 250 s. Suction-type mechanical stabilizer (Octopus, TS2000) was used to immobilize the target coronary artery. The distal anastomosis was completed with 7–0 or 8–0 polypropylene and standard techniques. LIMA was separately grafted to left anterior descending (LAD). SV was sequentially grafted to obtuse marginal (OM), diagonal and posterior descending artery (PDA) if needed. In some cases, SV was grafted to LAD because of poor flow of LIMA.

### Statistical analysis

Continuous variables are provided as median (quartile) or mean ± standard deviation (SD) based on the data dispersion. Classification variables are expressed as percentages. T-test is used to compare whether continuous variables obey normal distribution. If the variables are skew distribution, then non parametric Mann Whitney U test is used. Chi square test was used to compare the classified variables. Logistic regression analysis was used to determine the effect of IABP on postoperative outcome. Both unadjusted and multivariable adjusted models are applicable. All analyses were performed using statistical package R (http://www.r-project.org, R Foundation) and empower stats (http://www.empowerstats.com, X &amp; Y solutions, Inc., Boston, MA). A bilateral significance level of 0.05 was considered statistically significant.

## Result

The Table 1 showed the baseline characteristic of the patients. Univariate analysis showed that patients receiving IABP were more likely to have diabetes, myocardial infarction (MI), lower ejection fraction (EF), left ventricular diastolic dimension (LVDD), higher New York Heart Association class (NYHA), left internal mammary artery (LIMA) (Table 1).

**Table 1:** Baseline characteristic of patient population

	Control group (*N* = 132)	IABP group (*N* = 125)	*P* value
Age(y)	62.4 ± 8.4	62.6 ± 8.5	0.81
Male (%)	113 (85.6%)	96 (76.8%)	0.07
BMI	25.3 ± 3.8	25.3 ± 2.9	0.86
Smoker (%)	72 (54.5%)	65 (52.0%)	0.68
Hypertention (%)	73 (55.3%)	69 (55.2%)	0.98
Diabetes (%)	35 (26.5%)	47 (37.9%)	0.05
MI (%)	12 (9.1%)	35 (28.0%)	< 0.001
COPD (%)	2 (1.5%)	5 (4.0%)	0.22
PCI (%)	8 (9.6%)	12 (6.1%)	0.30
Cholesterol (mmol/l)	5.2 ± 9.3	4.1 ± 1.7	0.14
Creatinine (umol/l)	75.0 ± 19.0	79.9 ± 23.1	0.07
EF(%)	61.9 ± 7.4	52.5 ± 11.3	< 0.001
LVDD (mm)	51.2 ± 6.8	47.8 ± 4.4	< 0.001
NYHA class			< 0.001
II	112 (87.5%)	77 (62.6%)	
III	15 (11.7%)	46 (37.4%)	
IV	1 (0.78%)	0 (0%)	
Unstable angina	97 (73.5%)	91 (72.8%)	0.90
Carotid artery stenosis, n (%)	28 (21.5%)	16 (12.9%)	0.07
Stoke	1 (0.8%)	2 (1.6%)	0.53
Grafts, n			0.630
2	15 (11.4%)	16 (12.8%)	
3	96 (73.2%)	85 (68.0%)	
4	20 (15.2%)	24 (19.2%)	
LIMA	86 (65.6%)	65 (52.0%)	0.026

*MI* Myocardial infarction, *COPD* Obstructive pulmonary disease, *PCI* Percutaneous coronary intervention, *EF* Ejection faction, *LVDD* Left ventricular diastolic dimension, *NYHA* New York Heart Association, *LIMA* Left internal mammary artery

Table 2 showed the crude outcomes of the patients undergoing OPCAB. The 30-day mortality rate was 1.52% in the control group and 1.6% in the prophylactic IABP group (*P* > 0.05). The percent of conversion to CPB was 3.79% in the control group and 2.4% in the IABP group (*P* > 0.05). The incidence of perioperative MI was 9.85% in the control group and 5.6% in the IABP group (*P* > 0.05). The composite of the conversion to CPB, MI and mortality was 13.6% in the control group and 8.8% in the prophylactic IABP group (*P* > 0.05). There was greater incidence of prolonged ventilation (47.2% vs. 22.7%, *P* < 0.001) and longer hospital stay (11.25 ± 4.41 vs. 9.77 ± 3.84, *P* = 0.005) in IABP group.

**Table 2:** The crude outcomes of the patients

	Control group (N = 132)	IABP group (N = 125)	*P*-value
Conversion to CPB	5 (3.79%)	3 (2.40%)	0.522
Perioperative MI	13 (9.85%)	7 (5.60%)	0.204
Mortality	2 (1.52%)	2 (1.60%)	0.956
Composite end	18 (13.6%)	11 (8.8%)	0.221
Bleeding for reexploring	4 (3.03%)	3 (2.40%)	0.756
Dialysis	1 (0.76%)	3 (2.40%)	0.288
Ventilation time > 24 h	30 (22.7%)	59 (47.2%)	< 0.001
Hospital stay	9.77 ± 3.84	11.25 ± 4.41	0.005

*CPB* Cardiopulmonary bypass, *MI* Myocardial infarction, *Composite end* = Conversion to CPB + MI + Mortality

Table 3 demonstrated the adjusted outcomes after logistic regression analysis. IABP did not show favorable effect on the conversion to CPB (RR 0.63, 95%CI 0.05–7.89, *P* = 0.7211), perioperative MI (RR 0.69, 95%CI 0.22–2.12, *P* = 0.5163), mortality (RR 0.65, 95%CI 0.04–10.25, *P* = 0.7608) or the composite end of the conversion, MI and mortality (RR 0.63, 95%CI 0.23–1.74, *P* = 0.3747). There was greater incidence of prolonged ventilation in IABP after adjustment (RR2.16, 95%CI 1.12–4.18, *P* = 0.0221).

**Table 3:** The Adjusted outcomes of the patients

*IABP*	*Unadjusted*		*Adjusted*	
	RR (95%CI)	P	RR (95%CI)	P
Conversion to CPB	0.62 (0.15, 2.67)	0.5254	0.63 (0.05, 7.89)	0.7211
Perioperative MI	0.54 (0.21, 1.41)	0.2094	0.69 (0.22, 2.12)	0.5163
Mortality	1.06 (0.15, 7.62)	0.9562	0.65 (0.04, 10.25)	0.7608
Composite end	0.61 (0.28,1.35)	0.224	0.63 (0.23, 1.74)	0.3747
Bleeding for reexploring	0.79 (0.17, 3.59)	0.7569	0.94 (0.14, 6.21)	0.9503
Dialysis	3.22 (0.33, 31.38)	0.3139	0.65 (0.02, 25.87)	0.8173
Ventilation time > 24 h	3.04 (1.78, 5.20))	< 0.001	2.16 (1.12, 4.18)	0.0221

*CPB* Cardiopulmonary bypass, *MI* Myocardial infarction, *Composite end* Conversion to CPB + MI+ Mortality; *RR* Relative risk

There was no IABP-related mortality or limb ischemia.

## Discussion

The main finding of this study is that there is no significant difference in early prognosis between the IABP group and the control group. There was no significant difference between the two groups in terms of mortality and comprehensive indicators of myocardial infarction. In LMD patients receiving OPCAB, preventive IABP may not be necessary.

Left main coronary artery stenosis > 70% is one of the risk factors for operative death in patients undergoing CABG, although there is controversy in identifying high-risk patients [9]. Calcified ascending aorta and aortic root are more likely to occur in patients with LMD, which may lead to severe ischemia, and intractable ventricular fibrillation in case of displacement of heart during OPCABG [10]. The most severe complication of OPCABG is hemodynamic compromise or collapse, which usually leads to conversion to CPB [11].

Preventive IABP after high risk CABG is controversial. Christenson et al. [12] randomized 138 patients with a combination of at least 2 of the following risk factors: LVEF< 40%, unstable angina, redo, left main coronary stenosis≥70% to receive IABP preoperatively versus no preoperative IABP. Hospital mortality was 1.4% in treatment group and 15.9% in the control group, and there was significant difference. Other studies [13–17] also showed the effectiveness of prophylactic IABP in patients with high risks. IABP scores were established to identify patients at risk for low cardiac output syndrome who may benefit from selective implantation of IABP during CABG [18].

Other studies did not demonstrate the favorable outcomes in IABP groups. Holman et al. [7] compared 550 patients treated with preventive precut balloon pump with 550 patients not treated with preventive precut balloon pump. There was no significant difference in survival rate between patients treated with preventive precut balloon pump and those not treated with preventive precut balloon pump. However, compared with patients who did not receive balloon pump, survival patients who received balloon pump before incision had significantly shorter postoperative residence time (7 + 7.3 days) (8 + 6.2 days; *P* < 0.05). Emmert et al. [18] also reported that routine off-pump coronary artery bypass grafting is safe and feasible in high-risk patient with left main disease. In our treatment of OPCABG for patients with isolated LMD, LAD was usually grafted with LIMA before cardiac movement and examination of OM and PDA to avoid severe ischemia or hemodynamic collapse due to cardiac displacement. Since no significant difference in early outcomes were observed between IABP group and control group, LMD may be less dangerous than other high risks such as heart failure or low EF during OPCAB. Prophylactic IABP may be unnecessary in patients with LMD undergoing OPCAB.

It is reported that the postoperative stay time of patients receiving IABP is significantly shorter [7]. But in our study, the preventive IABP group had longer hospitalization time and a greater incidence of prolonged ventilation. The reason is not clear. We supposed that the degree of stenosis of left main artery in IABP group is larger, which may lead to more serious myocardial ischemia and need longer support or recovery. In our study, there was no IABP-related mortality or limb ischemia. Cohen et al. [19] reported the percentage of IABP-related mortality was 0.053%, major limb ischemia was 0.9% and severe bleeding was 0.9%. In our study, preoperative ultrasound examination on femoral artery and cautious insertion may help to reduce the incidence of IABP-related morbidity.

There are some limitations to our study. First, the small sample size and the nature of retrospective design weakens the strength of the paper. Second, only early outcomes were analyzed in our study. Third, single center experience needs to be testified in other units. More randomized clinical trials on this topic should be designed to evaluate the role of prophylactic IABP in LMD or other high risk patients in future.

## Conclusion

No significant difference in early outcomes was observed in hemodynamically stable patients with LMD between prophylactic IABP group and control group. Prophylactic IABP may be unnecessary in patients with LMD undergoing OPCABG.

## Data Availability

The data used to support the findings of this study are available from the corresponding author upon reasonable request.

## Abbreviations

IABP: Intra-aortic balloon pump
LMD: Left main disease
OPCABG: Off-pump coronary artery bypass grafting
BP: Blood pressure
RR: Relative risk
CI: Confidence interval
MI: Myocardial infarction
EF: Ejection fraction
CABG: Coronary artery bypass grafting
NYHA: New York heart association
LIMA: Left internal mammary artery
SV: Saphenous vein
LAD: Left anterior descending
OM: Obtuse marginal
PDA: Posterior descending artery; standard deviation (SD)
LVDD: Left ventricular diastolic dimension
COPD: Obstructive pulmonary disease
PCI: Percutaneous coronary intervention
EF: Ejection faction
CPB: Cardiopulmonary bypass
Composite end: Conversion to CPB + MI + Mortality
